# Development of efficient catharanthus roseus regeneration and transformation system using agrobacterium tumefaciens and hypocotyls as explants

**DOI:** 10.1186/1472-6750-12-34

**Published:** 2012-06-29

**Authors:** Quan Wang, Shihai Xing, Qifang Pan, Fang Yuan, Jingya Zhao, Yuesheng Tian, Yu Chen, Guofeng Wang, Kexuan Tang

**Affiliations:** 1 Plant Biotechnology Research Center, SJTU–Cornell Institute of Sustainable Agriculture and Biotechnology, Fudan-SJTU-Nottingham Plant Biotechnology R&D Center, School of Agriculture and Biology, Shanghai Jiao Tong University, 800 Dongchuan Road, Minhang District, Shanghai 200240, China

**Keywords:** *Catharanthus roseus*, *Agrobacterium tumefaciens*, Deacetylvindoline-4-*O*-acetyltransferase, Regeneration, Vindoline

## Abstract

**Background:**

As a valuable medicinal plant, Madagascar periwinkle (*Catharanthus roseus*) produces many terpenoid indole alkaloids (TIAs), such as vindoline, ajamlicine, serpentine, catharanthine, vinblastine and vincristine et al. Some of them are important components of drugs treating cancer and hypertension. However, the yields of these TIAs are low in wild-type plants, and the total chemical synthesis is impractical in large scale due to high-cost and their complicated structures. The recent development of metabolic engineering strategy offers a promising solution. In order to improve the production of TIAs in *C. roseus*, the establishment of an efficient genetic transformation method is required.

**Results:**

To develop a genetic transformation method for *C. roseus*, *Agrobacterium tumefaciens* strain EHA105 was employed which harbors a binary vector pCAMBIA2301 containing a report *β*-glucuronidase (*GUS*) gene and a selectable marker neomycin phosphotransferase II gene (*NTPII*). The influential factors were investigated systematically and the optimal transformation condition was achieved using hypocotyls as explants, including the sonication treatment of 10 min with 80 W, *A. tumefaciens* infection of 30 min and co-cultivation of 2 d in 1/2 MS medium containing 100 μM acetosyringone. With a series of selection in callus, shoot and root inducing kanamycin-containing resistance media, we successfully obtained stable transgenic regeneration plants. The expression of *GUS* gene was confirmed by histochemistry, polymerase chain reaction, and genomic southern blot analysis. To prove the efficiency of the established genetic transformation system, the rate-limiting gene in TIAs biosynthetic pathway, *DAT,* which encodes deacetylvindoline-4-*O*-acetyltransferase, was transferred into *C. roseus* using this established system and 9 independent transgenic plants were obtained. The results of metabolite analysis using high performance liquid chromatography (HPLC) showed that overexpression of *DAT* increased the yield of vindoline in transgenic plants*.*

**Conclusions:**

In the present study, we report an efficient *Agrobacterium*-mediated transformation system for *C. roseus* plants with 11% of transformation frequency. To our knowledge, this is the first report on the establishment of *A. tumefaciens* mediated transformation and regeneration of *C. roseus*. More importantly, the *C. roseus* transformation system developed in this work was confirmed in the successful transformation of *C. roseus* using a key gene *DAT* involved in TIAs biosynthetic pathway resulting in the higher accumulation of vindoline in transgenic plants.

## Background

**Figure 1:**
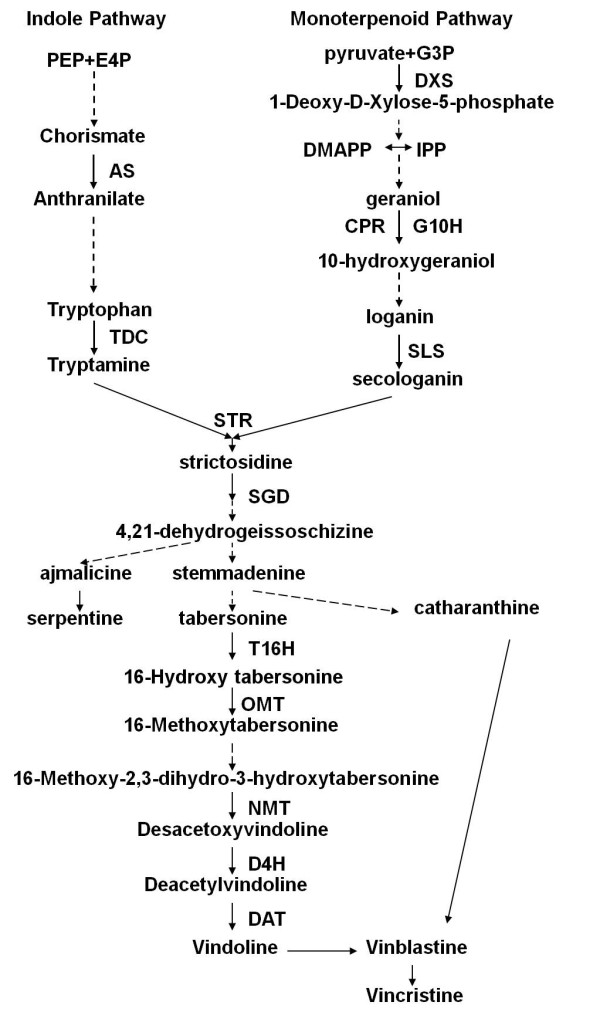
**Biosynthesis of *C. roseus *TIAs.** The abbreviations stand for anthranilate synthase (AS), tryptophan decarboxylase (TDC), geraniol-10-hydroxylase (G10H), cytochrome P450 reductase (CPR), secologanin synthase (SLS), strictosidine synthase (STR), strictosidine *β*-D-glucosidase (SGD), tabersonine-16-hydroxylase (T16H), 16-hydroxytabersonine-16-*O*-methyltransferase (16-OMT), minovincinine-19-*O*-acetyltransferase (MAT), desacetoxyvindoline-4-hydroxylase (D4H), deacetylvindoline-4-*O*-acetyltransferase (DAT), and Peroxidase (PRX). Broken arrows indicate multiple-step or uncharacterized enzymatic conversions.

Madagascar periwinkle (*Catharanthus roseus*) has become a model plant for secondary metabolism studies [1]. The medicinal plant *C. roseus* possesses a large number of terpenoid indole alkaloids (TIAs) among which over 130 compounds have been isolated and identified [2]. The production of the most valuable dimeric alkaloids vinblastine and vincristine are extremely low in wild-type *C. roseus* plant [3], and hard to be improved using cell suspension and hairy root cultures systems [4]. In addition, it is also difficult to synthesize TIAs by chemical methods due to their complicated structures [5]. Until now, the TIAs metabolic pathway (Figure 1) of *C. roseus* has become clearer. Vinblastine and vincristine are the most important antitumor bisindole alkaloids, which are derived from the coupling of vindoline and catharanthine monomers. The biochemical synthesis of the six-step enzymatic conversion of tabersonine to vindoline has been studied extensively [6]. The terminal step of vindoline biosynthesis is catalyzed by deacetylvindoline-4-*O*-acetyltransferase (DAT). The recent development of metabolic engineering strategy offers a promising solution to improve the TIAs production in *C. roseus*. Therefore, the establishment of effective regeneration and transformation system for *C. roseus* is required.

To date, genetic transformation of *C. roseus* has been mostly confined to hairy roots and suspension cells. *Agrobacterium rhizogenes-*mediated transformation involving productions of hairy (transgenic) roots in *C. roseus* had been reported [4,7,8]. However, the phenotypes of transgenic *C. roseus* plants transformed by *A. rhizogenes* are abnormal, such as shortened internodes, wrinkled leaves and abundant root mass [7]. Thus this kind of transgenic *C. roseus* plants is not suitable for the production of TIAs. Transgenic *C. roseus* cell suspension cultures transformed by either *Agrobacterium* infection or by particle bombardment had been established and studied intensively [4,9-13]. But these transgenic cells lines do not produce alkaloids in a stable manner and their ability to accumulate TIAs is gradually declined by long-term subculture [14]. Recently *A. tumefaciens*-mediated transformation was employed in *C. roseus*, the transgenic callus and plants were obtained respectively [15,16]. However, these transformation systems were not confirmed with other biochemical assays such as southern blot and high performance liquid chromatography (HPLC). To address these issues, in the present study we developed an *A. tumfaciens* mediated transformation and regeneration system of *C. roseus*. The stable regeneration plants were successfully acquired. To demonstrate this transformation system, *DAT*, an essential gene in TIAs biosynthetic pathway was overexpressed and the accumulation of vindoline was analyzed using HPLC in transformants.

## Results and discussion

### Optimization of transformation conditions for *C. roseus*

To develop an efficient system for producing transgenic *C. roseus* plants using *A. tumefaciens*, the association parameters were systemically investigated and optimized, including the concentration of *Agrobacterium* and acetosyringone, co-cultivation duration, and sonication condition. The detection was performed using the *A. tumefaciens*-mediated *GUS* gene transient expression. The transformation frequency was calculated as the number of kanamycin resistance plants/number of explants × 100.

Firstly, we assessed the *A. tumefaciens* density and duration during infection. The density of *A. tumefaciens* culture corresponding to OD_600_ = 0.5, 0.8 and 1.0 were chosen. These *A. tumefaciens* cultures were re-suspended to OD_600_ = 0.5 with liquid MS supplied with 100 μM acetosyringone. Then the hypocotyls were treated for 15, 30 and 45 min respectively. The results showed that the highest GUS transient expression (100%) and the relatively lower death rate for the explants (15%) were achieved using the OD_600_ = 0.8 of *A. tumefaciens* and the explants infection of 30 min (Table 1).

**Table 1:** **Influence of transformation factors on the frequency of transient *GUS *expression (%) in hypocotyls *of C. roseus***

**Factors**	**Variable**		**Transient expression rate (%)**^**a**^**(mean ± SE)**^**c**^	**Death rate (%)**^**a**^**(mean ± SE)**^**b**^
*A. tumefaciens* infection density(OD) and duration (min)	0.5	15	53.33 ± 3.33 c	10.00 ± 2.89 b
		30	78.33 ± 4.41 ab	11.67 ± 1.67 a
		45	80.00 ± 2.89 a	11.67 ± 3.33 a
	0.8	15	78.33 ± 3.33 b	15.00 ± 1.67 b
		30	100.00 ± 0.00 a	15.00 ± 2.89 b
		45	100.00 ± 0.00 a	21.67 ± 1.67 a
	1.0	15	73.33 ± 1.67 b	18.33 ± 3.33 c
		30	100.00 ± 0.00 a	46.66 ± 6.01 b
		45	100.00 ± 0.00 a	68.33 ± 4.41 a
Co-cultivation period (d)	1		76.67 ± 4.41 b	0.00 ± 0.00 c
	2		100.00 ± 0.00 a	5.00 ± 2.89 b
	3		100.00 ± 0.00 a	20.00 ± 5.77 a
Acetosyringone concentration (μM)	0		13.33 ± 3.33 c	0.00 ± 0.00 cd
	50		85.00 ± 2.89 ab	1.66 ± 1.66 bc
	100		100.00 ± 0.00 a	5.00 ± 2.89 b
	150		100.00 ± 0.00 a	46.67 ± 4.41 a

Transient expression of *GUS* gene was examined after co-culture. The data of death rates were collected after 10 d of co-culture. Hypocotyls were placed on MSCP1 medium.

^a^ Transient expression rate = number of GUS^+^(GUS-positive) explants/number of explants × 100.

^b^ Death rate = number of died explants/number of explants × 100.

^c^ Values represent means for 60 explants per treatment, replicated three times. Mean ± SE in each column followed by the same letter are not significantly different according to Tukey’s multiple comparison test (P ≤ 0.05).

Secondly, the effects of co-cultivation duration and acetosyringone concentration on transformation frequency were optimized separately. The co-cultivation with *A. tumefaciens* between 1 to 3 d and the acetosyringone concentration (0, 50, 100 and 150 μM) on T-DNA delivery were tested. The results showed that the highest frequency of GUS transient expression (100%) and the relatively lower death rate (5%) were obtained for the explants with 2 d co-cultivation and 100 μM of acetosyringone in co-culture medium in dark (Table 1).

**Figure 2:**
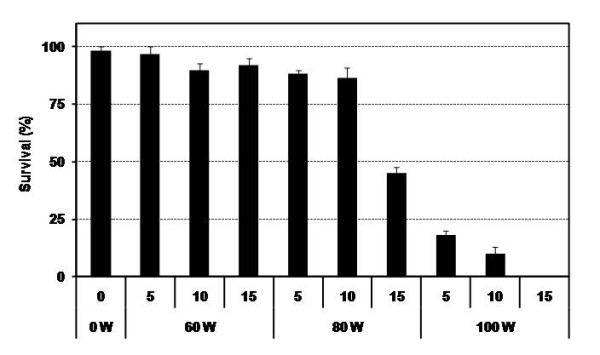
**Effect of sonication intensity and time on the survival rate of hypocotyls after 10 days in MSCP1 medium.** Survival rate of explants was evaluated at 10 days after co-cultivation. Only the green explants were taken into account, and the withered were thought as non-survival. Survival rate = number of green explants/number of total explants × 100.

Thirdly, the effects of sonication power and time on transformation frequency were investigated. *Agrobacterium* provides one of the main vehicles for introducing foreign DNA into plants based on its Ti-plasmid. However, one disadvantage of *Agrobacterium* transformation is the host’s specificity, which results in low efficiency in certain species [17]. Sonication-assisted *Agrobacterium*-mediated transformation (SAAT) offers an option to enhance *Agrobacterium* transient or stable transformation efficiency in different plant tissues [18]. The explanation might be the micro wounding formed by sonication on the surface and sub-surface layers of targeted tissue, which secrets more phenolic compounds and enhances the efficiency of transformation [19]. Up to now, the SAAT has been successfully employed to enhance transformation efficiency in several plant species, including kidney bean [20], radish [21], flax [22] and chickpea [23]. However, sonication alone has its negative effects on survival rate of the explants. So in this work the intensity and period of sonication were evaluated in order to seek the optimization of sonication application on *C. roseus* transformation. Before *A. tumefaciens* infection, *C. roseus* hypocotyls were transferred into liquid MS medium with 100 μM acetosyringone, and then sonicated with different power (0, 60, 80 and 100 W) and time (5, 10 and 15 min), respectively. The results are shown as Figure 2. After 2 d co-cultivation, we found that the transient expression of *GUS* stain was enhanced with the increase of sonication time and power (data not show). This result indicated that the transient transformation efficiency can be improved with the sonication treatment, which is accord with previous reports [12,16]. The growth of explants was further observed by transferring them to the MSCP1 culture medium (Table 2). The sonication power of 0 ~ 60 W and 0 ~ 15 min had no influence on the survival rate of these explants (Figure 2). However, the survival rate was decreased to 45% when the ultrasound treatment increased to 80 W and 15 min. Furthermore, when the sonication power arrived at 100 W, the survival rate of explants was decreased remarkably. Altogether, the sonication treatment of 80 W and 10 min can improve the transformation efficiency of *C. roseus* while keeping relatively high surviving rate (85%).

**Table 2:** The regeneration mediums used in this study

**Mediums**	**The components of the medium**
MSCP	MS + 150 mg/L casein hydrolysate + 250 mg/L L-proline + 30 g/L sucrose + 3 g/L gelrite
MSCP1	MSCP + 1.0 mg/L 2,4-D + 1.0 mg/L NAA + 0.1 mg/L ZT + 250 mg/L carbenicillin
MSCP2	MSCP + 5.0 mg/L BA + 0.5 mg/L NAA + 250 mg/L carbenicillin
MSCP3	MSCP + 1.75 mg/L BA + 0.55 mg/L IAA + 250 mg/L carbenicillin

Fourthly, the influence of kanamycin concentration on plant regeneration was assessed. The types of selectable markers and the selection pressure are very important factors for successful transformation [24]. The neomycin phosphotransferase gene which confers on the plant resistance to kanamycin is used in this protocol. Selection during callus induction and shoot initiation encourages regeneration of putative transgenic plants [25]. In the present study, three media (MSCP1, MSCP2 and MSCP3) (Table 2) supplied with different concentrations of kanamycin (Table 3) were prepared and used in sequence. Considering the growth of plant itself may strengthen resistance to kanamycin, the relatively higher kanamycin concentration was used in the media from MSCP1 to MSCP3.

**Table 3:** Experimental design for kanamycin concentration optimization

**Treatment**	**Kan concentration of MSCP1 (mg/L)**	**Kan concentration of MSCP2 (mg/L)**	**Kan concentration of MSCP3 (mg/L)**
K1	40	50	70
K2	40	50	90
K3	40	50	110
K4	40	70	70
K5	40	70	90
K6	40	70	110
K7	40	90	90
K8	40	90	110

**Figure 3:**
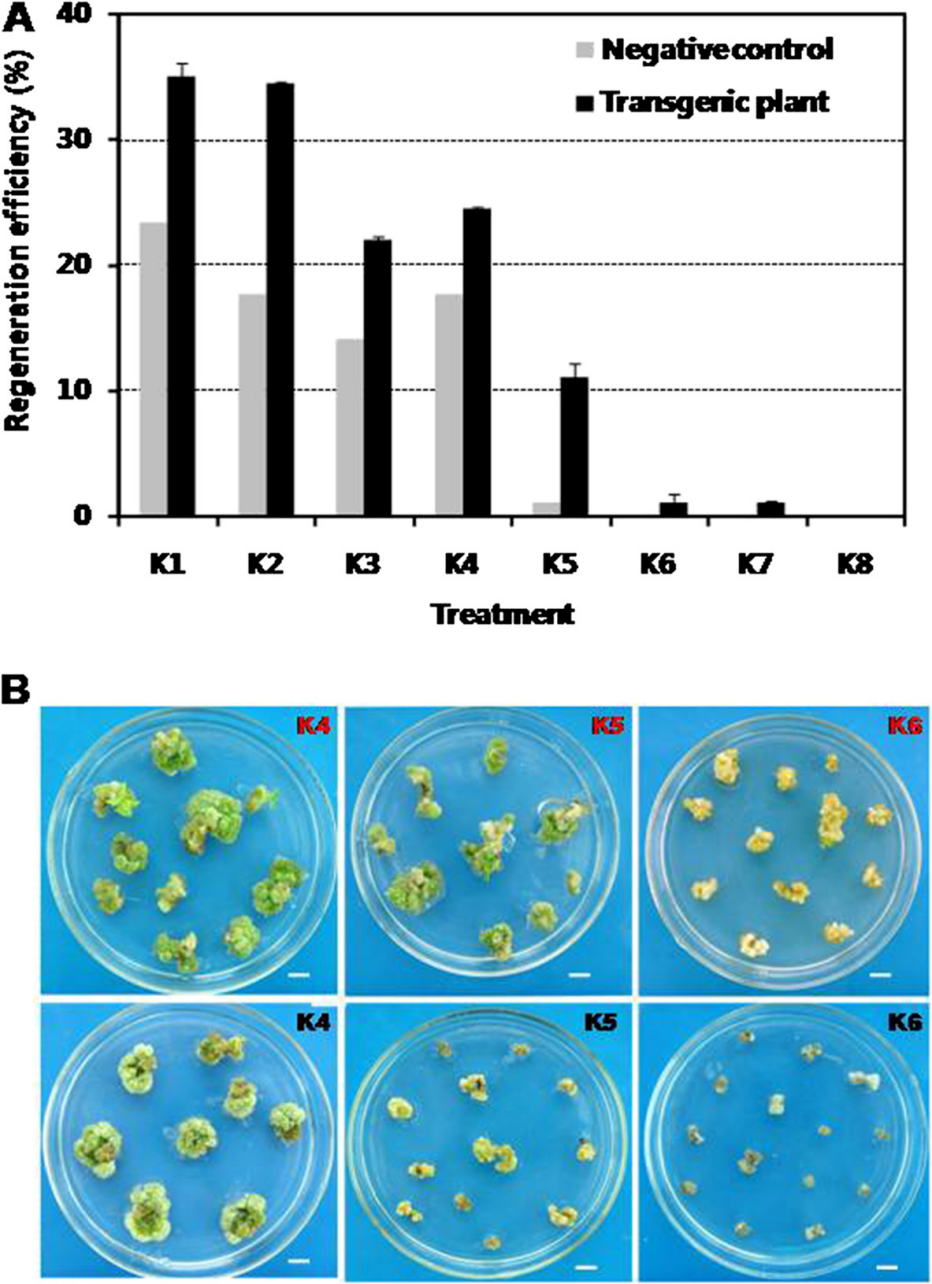
**Influence of kanamycin concentration on the regeneration of *C. roseus *in shoot elongation (MSCP3) medium. A** K1-K8 represent culture conditions with different kanamycin concentrations. Regeneration efficiency = number of regeneration explants/number of total explants × 100. **B** The red words represent *Agrobacterium*-mediated transformation with resistant strains, and the black words represent *Agrobacterium*-mediated transformation with non-resistant strains. The pictures were taken at the 30^th^ day after co-culture in shoot elongation medium. Scale bar = 0.9 cm.

From Figure 3A, the regeneration frequency of transformed and non-transformed explants was declined with the increase of kanamycin concentration. With the kanamycin concentration of 40 mg/L in MSCP1 and 50 mg/L in MSCP2 (K1-K3 treatment), the differentiation of non-transformed explants was inhibited comparing to transformed explants. But the regeneration frequency of non-transformed explants is still from 13% to 23%. When the kanamycin concentration higher than 70 mg/L in MSCP2 and 90 mg/L in MSCP3 (K5-K8 treatment), the regeneration of non-transformed explants was almost completed inhibited. The representative images are shown as in Figure 3B, which exhibit the contrast of growth state between non-transformed and transformed explants. At last, 40 mg/L in MSCP1, 70 mg/L in MSCP2 and 90 mg/L in MSCP3 kanamycin (K5 treatment) were used for selecting transformed plants due to the relatively high regeneration efficiency of transformed explants (11%) and low regeneration efficiency of non-transformed explants (1%).

### Regeneration of transgenic *C. roseus*

**Figure 4:**
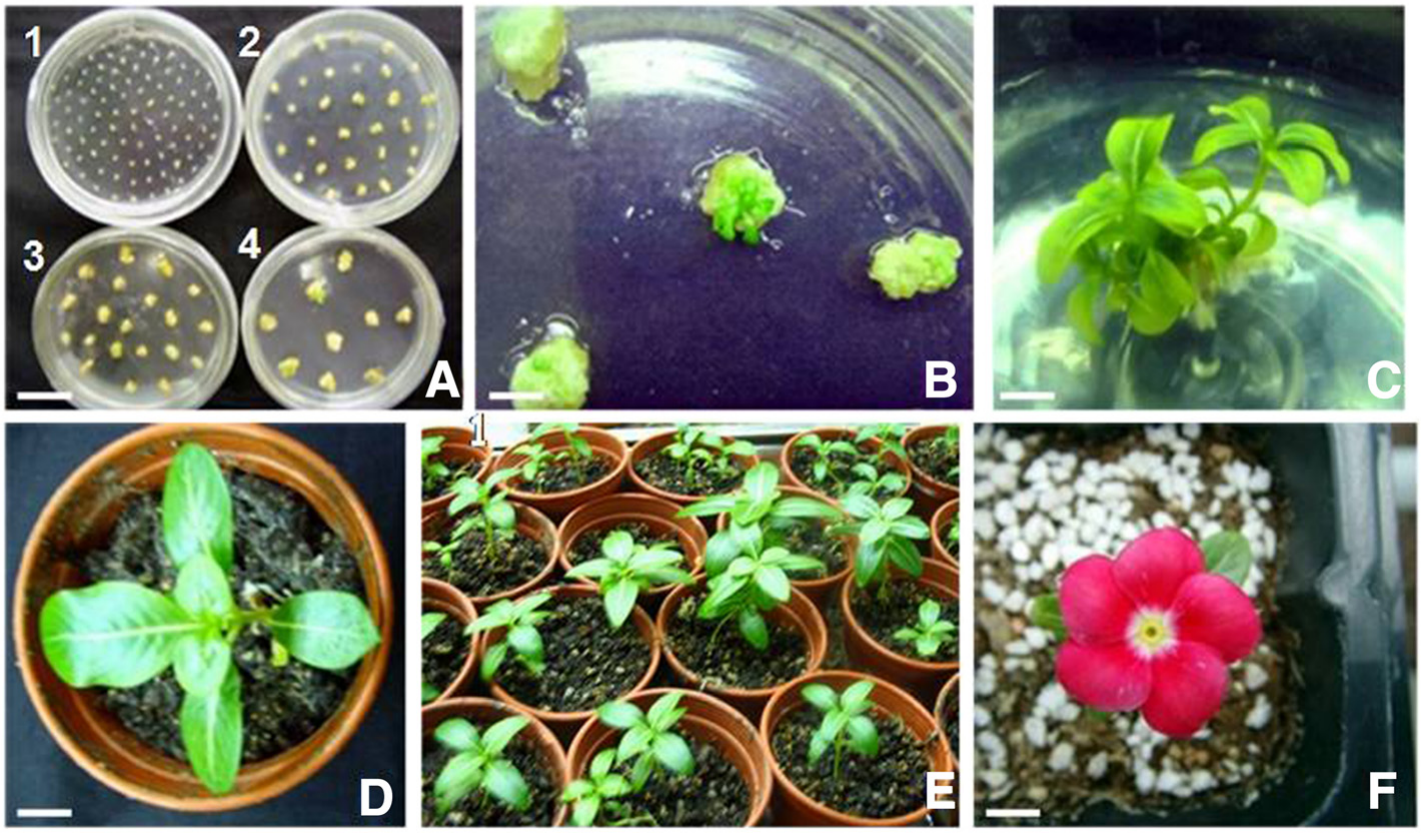
**Regeneration and transformation of *C. roseus. *A** The regenerative phase of *C. roseus* (1: Sowing in MS medium; 2: Callus induction in MSCP1 medium; 3, 4: Shoots initiation in MSCP2 and MSCP3 medium separately). **B** Shooted plantlet. **C** Rooted plantlet. **D, E** Transgenic plantlets in soil. F Flower seedlings. Scale bar = 3 cm in **A**, 1 cm in **B, F**, 2 cm in **C** and 1.5 cm in **D**.

**Figure 5:**
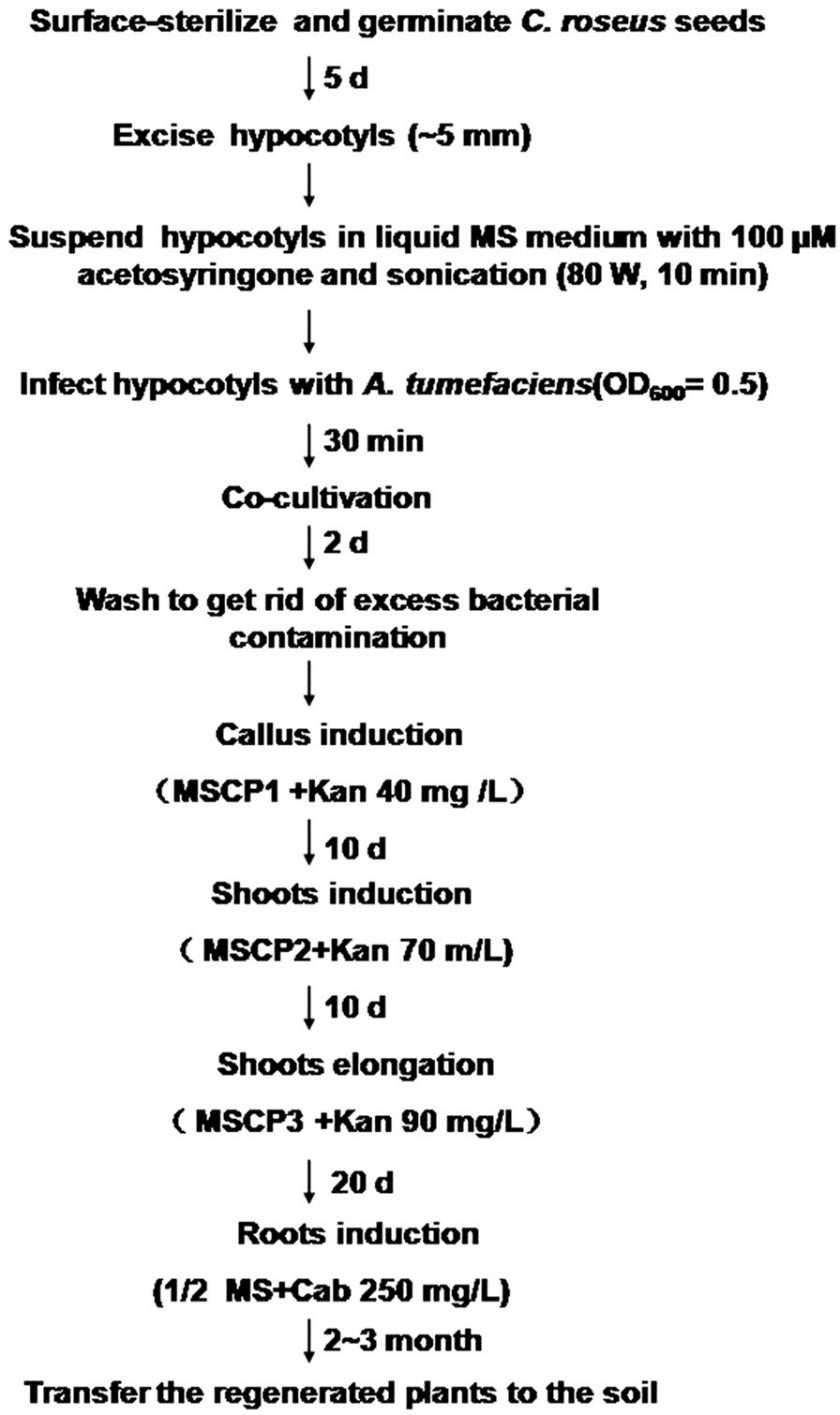
**The procedure of *C. roseus *transformation and regeneration using hypocotyls as explants**.

Using hypocotyls as explants, callus induction, shoot initiation and root initiation media containing kanamycin were employed in sequence. After *Agrobacterium*-mediated transformation and 2 d co-cultivation in dark, transgenic calli were induced by growing the hypocotyl explants for 10 d in selective medium (MSCP1) with kanamycin. Then the induced transgenic calli were subjected to additional two rounds of selection on medium (MSCP2 and MSCP3) containing higher levels of kanamycin (Figure 4A), which helps to eliminate false-positive plants. In this experiment, we found most explants became brown gradually and died except the putative transformants. The green shoots of putative transformants that appeared in 40 d (Figure 4B) were transferred onto roots induction medium (Figure 4C). At last, the rooted plants grew normally after transplanted to soil in the green house (Figure 4D, E, and F). It took 3 ~ 4 months using hypocotyls as starting materials from inoculation with *A. tumefaciens* to transplant to soil. The procedures of *C. roseus* transformation were showed in Figure 5.

### Molecular characterization of transgenic *C. roseus* plants with *GUS*

**Figure 6:**
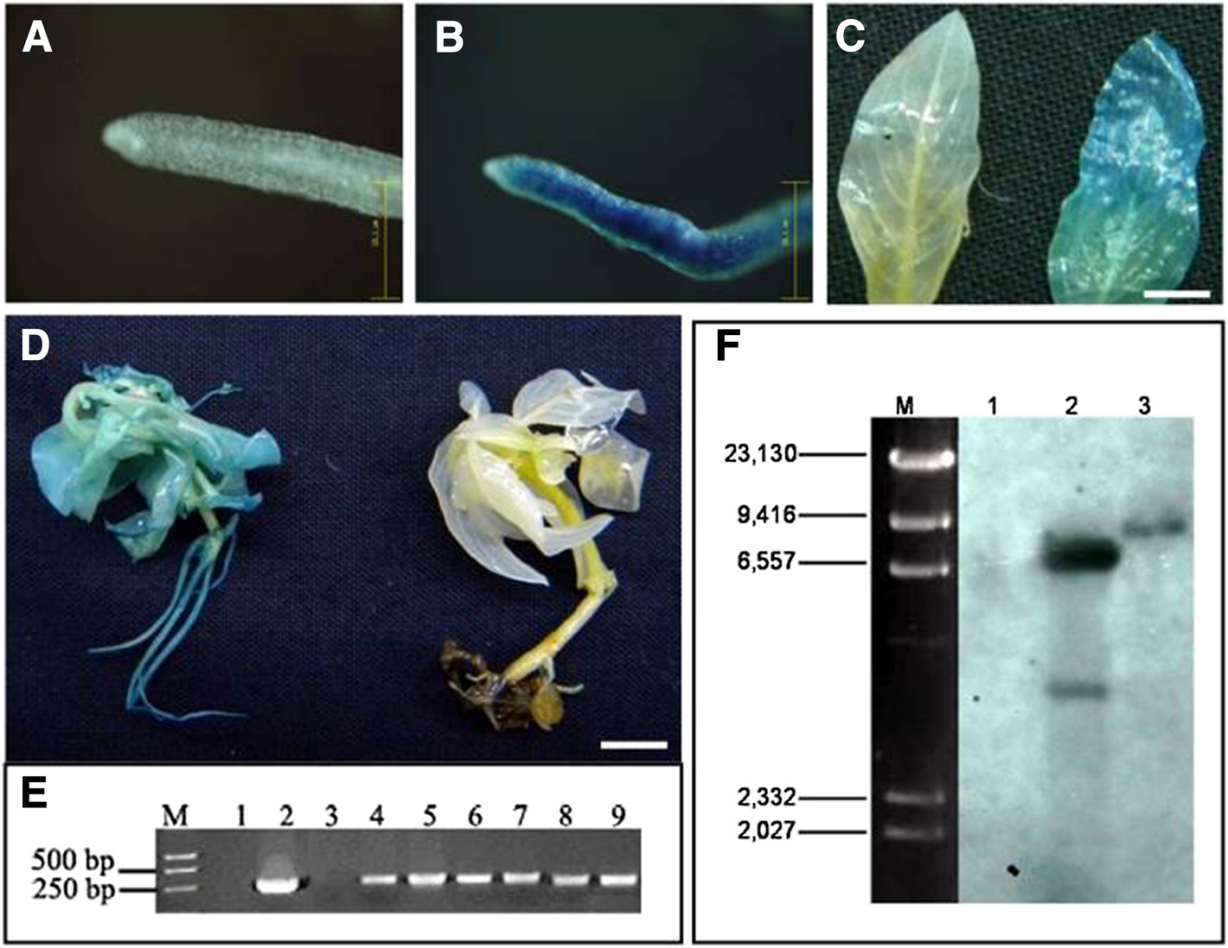
**Analysis of transgenic GUS *C. roseus*. A-D** Histochemical GUS expression in transgenic *GUS* plants of regenerated *C. roseus.***A**, C-left, D-right: non-transformed control; **B**, C-right and D-left: transgenic plant. Scale bar = 25.1 μm in **A** and **B**, 0.5 cm in **C** and 1 cm in **D**. **E** Detection of *GUS* gene in kanamycin-resistant *C. roseus* plants using PCR. M, DL2000 DNA ladder (Maker from TaKaRa); 4–9, regenerated transgenic plant; 1, DNA from non-transformant (negative control); 2, pCAMBIA2301 was used as template (positive control); 3, H_2_O as template (negative control). **F** Southern-blot analysis of transgenic *C. roseus* plants. 1 is untransformed control plant; 2 and 3 are the DNA from two independed transformants digested by *Hind*III, respectively. M, λ-*Hin*d III Marker.

To confirm the transformation events, histochemical analysis of GUS activity was carried out in putative kanamycin resistant transgenic lines. GUS positive blue coloration was visibly detected in all transgenic tissues stained with X-gluc reagent (Figure 6B, C-right and D-left). However the GUS staining was not detected in the wild type plants which were neither sonicated nor transformed with *Agrobacterium* (Figure 6A, C-left and D-right).

To determine the presence and the integration of transgene in kanamycin resistant plants, PCR and DNA blot hybridization were performed with genomic DNA. First the putative transgenic plants were screened by PCR using *GUS* gene-specific primers to detect the presence of target gene in host. The representative results are shown as in Figure 6E. It indicates the 400 bp fragments of *GUS* genes in kanamycin-resistant transformed plants were amplified, which is the same as positive control. No specific amplification product was detected in non-transgenic control plants.

The transgenic plant integrated with foreign gene was further verified by southern blot with the fragment of *GUS* as probe. The genomic DNA was obtained from two independent PCR-positive and GUS-positive transgenic *C. roseus* plants. The southern results indicate that the T-DNA was inserted into genome of both transgenic plants. One transgenic plant has a single copy and another has two copies, while no signal is detected in the untransformed control (Figure 6F).

Altogether, the results of molecular analyses confirm that these regenerated plants are stably integrated with *GUS* gene. In this study we obtained 16 putatively transformed plants by transformed the explants three times using same method. The percentage of explants producing regenerates was 19% and the frequency of explants producing independent transgenic plants was established at 11%.

### Analysis of transgenic *C. roseus* plants with overexpressed *DAT*

**Figure 7:**
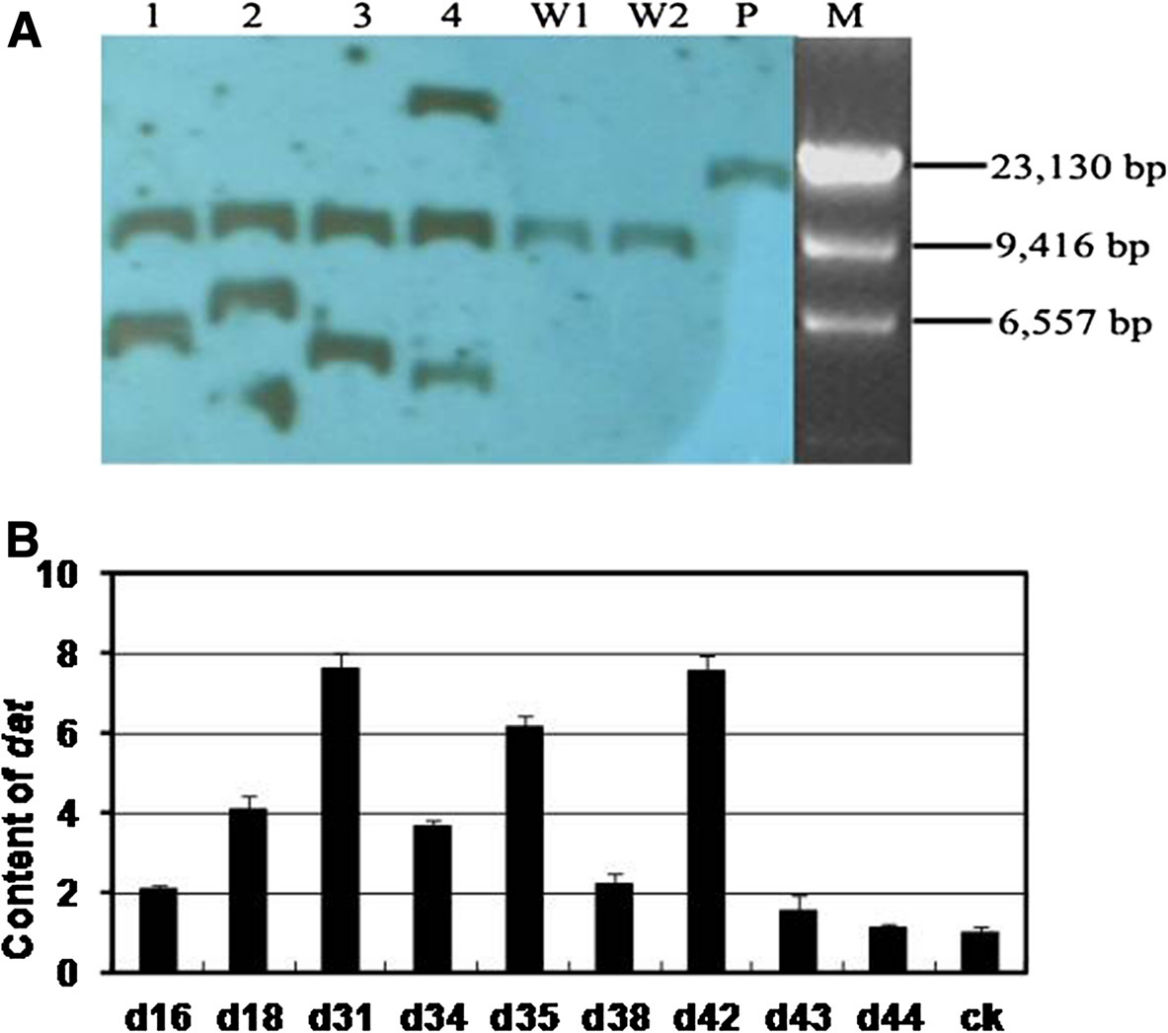
**Analyses of DAT expression independent transgenic *DAT *C*. roseus *using southern-blot and real-time PCR. A**. Southern blot analysis of four randomly selected *DAT* transformants. Genomic DNA of transformants (1–4), the wild-type DNA (W1, W2) and the plasmid P2301 + *DAT* (P) digested with *BamH*I were used in southern blot analysis. M, λ-*Hin*d β Marker. **B**. Real-time PCR analysis for the expression level of *DAT* gene in kanamycin-resistant *C. roseus* plants regenerated by transformation with pCAMBIA2301::p35s-*DAT*-nos. d16-d44, *DAT* transgenic plants; ck, average content for 15 control plants. The experiments were repeated at least 8 times and the same results were obtained.

In order to validate the established transformation system, the *DAT* was over expressed in *Catharanthus* plants using the same transformation procedure. 9 independent transgenic plants were confirmed by southern blot and/or PCR analyses (Figure 7). With the fragment of *DAT* as probe, the result of southern blot (Figure 7A) show that the transformants contained 1 to 2 more copies of *DAT* gene than the negative control.

The *DAT* mRNA level was analyzed using real-time PCR in 9 independently *DAT* transformed *C. roseus* plants. The results reveal that the expression level of *DAT* was increased in all the transgenic plants (Figure 7B). Especially for d31, d35 and d42 transgenic plants, the expression of *DAT* was significantly increased by 7.64, 6.14 and 7.58-fold respectively.

**Figure 8:**
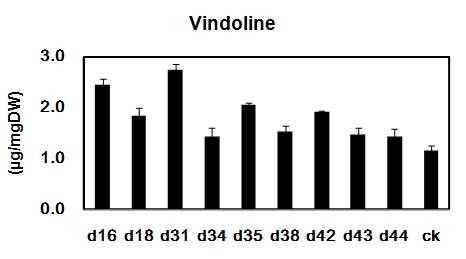
**Vindoline content of transgenic and untransformed *C. roseus *plants determined by HPLC analysis d16-d44, *DAT *transgenic plants; ck, average content for 15 control plants**.

At last, the yield of vindoline was determined by HPLC in *DAT* overexpressed transgenic plants. The pCAMBIA2301 transgenic and wild type plants were served as negative controls. With *DAT* overexpression, the results show that the production of vindoline was increased in all transformants (Figure 8). The amount of vindoline was 1.42 ~ 2.72 μg/mg (DW) in transgenic plants with over-expressed *DAT,* and about 1.15 μg/mg (DW) in both pCAMBIA2301 and wild-type plants. Especially for the d31, the accumulated vindoline was 2.72 μg/mg (DW), 2.4-folds than that of controls. This finding is consistent with the real-time PCR results, which suggests that *DAT* expression is associated with the accumulation of vindoline. However, further investigations will be needed involving more number of transformed plants to decipher the precise role of *DAT* genes in the regulation of TIAs pathway in *C. roseus*.

Because the transformation of *C. roseus* at whole plant level had no report before, the functions of genes in TIAs were investigated using hair root or suspension cell transformation so far. Here the established transformation system provides a potential possibility to investigate the effect of gene expression upon the alkaloids yields on *C. roseus* whole plant, and would contribute to the successful modification of the medicinal plants for higher natural product yields.

## Conclusions

Here we report an *Agrobacterium*-mediated transformation and regeneration system for *C. roseus.* The parameters influencing the transformation frequency are systematically investigated, including the concentration of *Agrobacterium* and acetosyringone, co-cultivation duration, sonication condition and selection pressure of kanamycin. The results show that the transformation frequency arrived at 11%. In order to validate the established transformation system, the key gene, *DAT,* in TIAs biosynthetic pathway was overexpressed. The HPLC results reveal that the production of vindoline was enhanced in transgenic plants with *DAT* overexpression.

In conclusion, all the results obtained show that the *C. roseus* transformation protocols developed in this work has great potential to be used in the discovery of genes function in TIAs biosynthetic pathway, in addition to improve the productions of TIAs.

## Methods

### Plant material

Seeds of *C. roseus* cultivar Pacifica Cherry Red were purchased from PanAmerican Seed Company (USA). Seeds were sterilized with 75% (v/v) ethanol for 1 min and 10% (v/v) NaClO for 10 min, and then were washed three times with sterile distilled water. Finally, seeds were transferred onto Petri plates containing MS [24] basal medium. Cultures were germinated 16 h light and 8 h dark photoperiod at 25 ± 2°C. Hypocotyls, about 5 mm, were excised from 5 d-old germinated seedlings.

### *A. tumefaciens* strain and vector used for transformation

For transformation, *A. tumefaciens* strain EHA105 harbouring the binary pCAMBIA2301 (CAMBIA Company, Australia) was used for transformation. The vector contains a *GUS* reporter gene and a selection marker gene *NPTII* (neomycin phosphotransferase gene conferring resistance to kanamycin) which are inserted between the CaMV35S promoter and the *A. tumefaciens nos* terminator separately. An intron inside the coding sequence is included in the *GUS* reporter gene to ensure that expression of glucuroidase activity is derived from eukaryotic cells.

To get fresh cells, a single colony of *A. tumefaciens* with pCAMBIA2301 vector was inculcated in liquid Luria Bertani (LB) medium containing 100 mg/L kanamycin and 100 mg/L rifampicin (Sigma, USA), and grown at 28°C for 36 h with shaking (150 rpm). The initial culture was diluted 1:1000 with liquid LB medium and grown on a shaker (250 rpm) until the OD_600_ reached to 0.5, 0.8 and 1.0. Then the cells were centrifuged (2,000 × g, 10 min) and the supernatant was removed. The bacterium was re-suspended in liquid MS medium containing 100 μM acetosyringone and the OD_600_ was adjusted to 0.5. At last, the bacterium was shaken (100 rpm) again for 2 h in dark at 28°C.

### Genetic transformation and co-cultivation

The explants of *C. roseus* were immersed in liquid MS medium with 100 μM acetosyringone in tissue culture tubes. Then these tubes were sonicated for 5/10/15 min (40 Hz, 0/60/80/100 W) with the sonicator DL-60D (Shanghai hengxin, China) separately. After that, the explants were transferred into pre-sterilized flasks containing bacterial suspension, and were shaken gently at 25 rpm for 30 min at room temperature. Explants were then blot-dried with sterile paper towels and transferred onto petri dishes containing 1/2 MS medium with 100 μM acetosyringone. The co-cultivation period was 1 ~ 3 d in the dark at 28°C.

### Plant regeneration

After co-cultivation, the explants were transferred into MSCP callus induction medium containing 1.0 mg/L 2, 4-D, 1.0 mg/L NAA, 0.1 mg/L ZT, 250 mg/L carbenicillin and 40 mg/L kanamycin (MSCP1 medium) for 10 d. Then the explants were transferred to the MSCP medium supplemented with 5.0 mg/L 6-BA, 0.5 mg/L NAA, 250 mg/L carbenicillin and 70 mg/L kanamycin (MSCP2 medium), which was effective on shoot initiation for 10 d. The resulting explants were transferred to shoot elongation MSCP medium supplemented with 1.75 mg/L 6-BA, 0.55 mg/L IAA, 250 mg/L carbenicillin and 90 mg/L kanamycin (MSCP3 medium) for 20 d, and subcultured every week. The elongated shoots were separated and transferred to root initiation medium (1/2 MS) containing 250 mg/L carbenicillin for 1 ~ 2 months. The regeneration seedlings were removed from the growth cabinet and were transferred to gardening soil. All the culture conditions were performed as pre-described for the plant seeding.

### Analysis of putative transformants

Histochemical GUS activities in leaf and root segments of the control and putatively transformed plantlets were investigated according to Jefferson [26]. Tissues were immersed in a buffer containing 2 mM X-Gluc, 50 mM phosphate, 50 mM potassium ferrocyanide and 5% Trition X-100 at pH 7.0. The reaction mixture was placed in a mild vacuum for 10 min, and then incubated overnight at 37°C. Tissues containing chlorophyll were repeatedly soaked in 95% ethanol until chlorophyll was removed. Transient expression of *GUS* gene was examined after 5 d of co-cultivation, while stable expression of the reporter gene was scanned after the *C. roseus* regeneration plants were transplanted to greenhouse for 3 months.

Genomic DNA was isolated from the young leaves of putative transgenic plants using CTAB method [27]. Putative transgenic plants were initially screened by PCR using the *GUS* gene-specific primers GUS FI (5^′^-GGGTGAAGGTTATCTCTATGAAC-3′) and GUS RI (5′-CACTGATACTCTTCACTCCACAT-3′) to detect the presence of target gene in the host.

The integration of the *GUS* gene in the transgenic *C. roseus* was examined by southern hybridization. Approximately 50 μg of genomic DNA per sample was digested with *Hind*III which was unique site in the plasmid. Then the digested DNA were fractionated by 1.0%-agarose-gel electrophoresis, transferred onto a positively charged Hybond-N^+^ nylon membrane (GE Heathcare, USA), and hybridized with an alkaline-phosphatase-labelled partial cDNA sequence of *GUS*. The probe (402 bp) was generated by PCR with primers GUS FII (5′-CAGTCTTACTTCCATGATTTCTTTA-3^′^) and GUS RII (5′-AGTAAAGTAGAACGGTTTGTGGTTA-3^′^). Hybridization and signal detection were performed using Amersham Gene Images AlkPhos Direct Labelling and Detection System (GE, UK). The hybridized signals were visualized by exposure to Fuji X-ray film at room temperature for 4 h.

In this work, the parameters of *A. tumefaciens* cell density, acetosyringone concentration, sonication condition and kanamycin concentration were investigated. Each treatment has three flasks replicates and each flask contains at least 20 explants. The data was analyzed with SAS software. The values are expressed as the mean of triplicate and analyzed by Duncan's New Multiple Range Test (DNMRT) with P value of 0.05.

### Validation of the established transformation system

After 3 months of the regenerated *C. roseus* growing in greenhouse, three pair leaves at the top of the transgenic *C. roseus* plants and control were collected and mixed, which were used for the analysis of real-time PCR and HPLC. *DAT* cDNA was cloned by RT-PCR using RNA extracted with Plant (Leaves) Total RNA Isolation Kit (Watson Biotechnologies, Inc, Shanghai, China). In RT-PCR, DAT-F (5^′^-CCCATGGGATGGAGTCAGGAAAAATATCG-3′, with *Nco*I site) and DAT-R (5^′^-CGGTAACCTTAATTAGAAACAAATTGAAGTAGC-3^′^, with *BstE*II site) were used as the forward and reverse primer respectively, and the parameters used during PCR reaction were as follows: 94°C, 5 min; 32 cycles: 94°C, 30 s; 52°C, 30 s, 72°C, 30 s; 72°C, 5 min. The amplified fragment was cloned into pMD18-T vector (TakKaRa, Japan) and sequenced. After confirmation by sequencing, the pMD18-DAT was double-digested by *Nco*I and *BstE*II, and then ligated into the *Nco*I and *BstE*II sites of pCAMBIA2301. The resultant plasmid *pCAMBIA2301-35 S::DAT::NOS* was then transferred into *A. tumefaciens* strain EHA105, and the resulting strains were used in the transformation of *C. roseus.* Transgenic *DAT C. roseus* plants were obtained using the same transformation procedure.

Southern blot analysis was carried out using a biotin-labelled 335 bp *DAT* fragment (DAT FII: 5'-GCTATTGTTCAACTAAGTCAT-3' and DAT RII: 5'-GCAGTCAAAACCTCTACTCGAG-3') as probe. Genomic DNA of randomly selected transgenic plants and a control plant were digested with *BamH*I. The probe labelling and hybridization was also performed as previously described. The expression level of *DAT* was detected by real-time PCR in which the detail procedures were shown as in our previous study [28]. Briefly, the specific primers for their corresponding genes were analyzed, which include DAT-FIII (5'-CTTCTTCTCATCACGTACCAACTC-3') and DAT-RIII (5'-ATACCAAACTCAACGGCCTTAG-3') for *DAT* gene, and Rps9-F (5'-TCGCAACTATGGTAAGACCT-3’) and Rps9-R (5'-CTGTTCATCCTCCTCAAAAG-3') for Rps9 gene. The cDNA for real-time PCR was synthesized from RNA samples using Prime Script^TM^ Reverse Transcriptase Reagent with oligo (dT) as primer according to the manufacturer’s instruction (TaKaRa, Japan). The real-time PCR analysis was performed in Peltier Thermal Cycler PTC200 (Bio-Rad, USA) using the cDNAs as templates. SYBR Premix Ex Taq (TaKaRa, Japan) was used in PCR reactions to quantify the amount of dsDNA. The relative Ct, the threshold of cycle value, was used to estimate the initial amount of template in reactions.

To prepare the samples for HPLC, the young leaves of transgenic *C. roseus* were dried at 45°C for 48 h and pulverized in a mortar [29]. Then 200 mg of each sample were immersed with 1 mL methanol for 10 min. The tubes were dipped into ultrasonic bath with the power of 80 W for 60 min. The mixture was centrifuged at 12,000 rpm for 10 min and the supernatant was filtered with 4 μm filter membrane. The extracts were stored at 4°C.

For HPLC analysis, the commercialized vindoline (Sigma-Aldrich, USA) was prepared at a concentration of 1 mg/L in methanol and used as standard [30]. The HPLC analysis was performed using a Sapphire-C18 (4.6 mm × 250 mm, 5 μm) column at a column temperature of 35°C and Hitachi L-2000 series HPLC system. This system consists of an L-2000 Organizer, an L-2130 Pump, an L-2200 AutoSampler, an L-2301 Column Oven and an L-2455 Diode Array Detector. The injected samples (10 μL) were detected at 220 nm by L-2455 Diode Array Detector. The mobile phase (acetonitrile and diethylamine buffer solution; 1:1) was used at a constant flow rate of 1 mL per min. The UV absorbance of standards and alkaloids were acquired. At last, the amount of vindoline was determined by using regression equation of calibration curve [29]. The TIAs level was determined by the areas of peak in chromatographic profile at 14.41 min for vindoline.

## Competing interests

Do you hold any stocks or shares in an organization that may in any way gain or lose financially from the publication of this manuscript, either now or in the future? If so, please specify. No.

Do you hold or are you currently applying for any patents relating to the content of the manuscript? Have you received reimbursements, fees, funding, or salary from an organization that holds or has applied for patents relating to the content of the manuscript? If so, please specify.

We have applied a patent regarding the *Catharanthus roseus* transformation method. We do not receive reimbursements, fees, funding from an organization (Shanghai Jiao Tong University, SJTU, the inventor's working institution) that applied for patent relating to the content of the manuscript, but only receive salary. Do you have any other financial competing interests? If so, please specify. Non-financial competing interests. No.

Are there any non-financial competing interests (political, personal, religious, academic, ideological, intellectual, commercial or any other) to declare in relation to this manuscript? If so, please specify. No.

## Authors’ contributions

QW performed all experiments, data analysis, and the manuscript preparation. SX took part in the experiments of Southern blot. QP, FY, YT, JZ and GW took part in the experiments of tissue culture and optimization of the transformation parameters. YC provided technical support of HPLC. KT supervised the study. All authors have read and approved the final manuscript.
